# Evaluating the potential of ChatGPT for patient identification in clinical breast cancer trials

**DOI:** 10.1177/20552076251389325

**Published:** 2025-11-20

**Authors:** Annika Krückel, Peter A Fasching, Oliver Schleicher, Julia Gocke, Lena Brückner, Katharina Seitz, Lothar Häberle, Felix Heindl, Carolin C Hack, Matthias W Beckmann, Julius Emons

**Affiliations:** 1 Department of Gynecology and Obstetrics, 27168 Universitätsklinikum Erlangen, Friedrich-Alexander-Universität Erlangen-Nürnberg (FAU), Erlangen, Germany; 2 Comprehensive Cancer Center Erlangen-European Metropolitan Area of Nuremberg (CCC ER-EMN), Erlangen, Germany; 3 Bavarian Cancer Research Center (BZKF), Erlangen, Germany; 4 Biostatistics Unit, Department of Gynecology and Obstetrics, Universitätsklinikum Erlangen, Friedrich-Alexander-Universität Erlangen-Nürnberg (FAU), Erlangen, Germany

**Keywords:** Artificial intelligence, oncology, clinical trials, women's health, cancer

## Abstract

**Objective:**

Growing complexity of oncological treatment is reflected in the requirements for current clinical trials, challenging clinical sites with recruiting suitable participants. This cross-sectional study evaluates the potential of artificial intelligence (AI), based on the example of ChatGPT-4.0, in identifying suitable study participants among patients with breast cancer, utilizing real-world tumor board data.

**Methods:**

ChatGPT-4.0 was trained on six fictitious study protocols for patients with breast cancer, mimicking real-world clinical trial scenarios. Anonymized data from 124 tumor board registrations from January 2024 were submitted to the AI to determine eligibility for study participation. A clinician control group also assessed the patients’ eligibility. The evaluations of ChatGPT-4.0 and the medical professionals were benchmarked against an expert-validated reference standard. Sensitivity and specificity were calculated for the AI as well as for each member of the control group.

**Results:**

Overall, among the 124 tumor board registrations, 19 patients met eligibility criteria for at least one study. Both AI and clinicians reliably excluded ineligible patients (high specificity), but sensitivity varied. ChatGPT-4.0 proved especially ineffective at screening for neoadjuvant trials, whereas medical professionals showed better, but heterogeneous performance. Team-based assessment identified nearly all eligible patients, underscoring the value of collaborative decision making.

**Conclusion:**

While model performance was limited by simplified input data and a small single-center cohort, the results suggest that ChatGPT-4.0, in its current form, is not yet suitable as a stand-alone tool for patient identification in clinical breast cancer trials. To ensure accurate and efficient recruitment, the involvement of a multiprofessional team remains essential. Ongoing model refinement and access to larger, more detailed datasets may enhance the future utility of AI systems in clinical trial screening.

## Objective

Gynecological oncology is witnessing an unprecedented increase in complexity due to the rapid evolution of treatment modalities and the growing heterogeneity of patient populations. Breast cancer represents the most prevalent malignancy among women globally, with approximately 2.3 million new cases reported in 2022.^1^ The disease comprises a wide range of subtypes with substantial differences in tumor biology, necessitating distinct therapeutic strategies.^2,3^ In recent years, a plethora of novel pharmaceuticals has undergone evaluation in clinical trials, progressively fostering the development of increasingly personalized treatment regimens. In the past decade, numerous drugs for targeted therapy in breast cancer have been approved. These include cyclin-dependent kinases 4 and 6 (CDK4/6) inhibitors, poly(adenosine diphosphate [ADP]-ribose) polymerase (PARP) inhibitors, phosphatidylinositol 3-kinase (PI3K)/protein kinase B (Akt) pathway inhibitors, immune checkpoint inhibitors, antibody drug conjugates, as well as an orally available selective estrogen receptor degrader (SERD).^4–15^ The deployment of such therapies requires not only the classification of patients according to traditional TNM (tumor node metastasis) tumor staging, therapy setting, and prior therapies, but also an understanding of the tumor's molecular features and, if applicable, patient-specific characteristics such as pathogenic germline variants.^4–14^ The complexity of gynecological oncology is reflected in the requirements of current clinical trials, where screening for suitable participants based on genetic, molecular, and clinical profiles, demands tools that can manage vast arrays of data efficiently and accurately. Manual screening of patients for clinical studies in the field of breast cancer remains a labor-intensive and time-consuming process. Clinical staff must review extensive and often heterogeneous patient records to identify eligibility, which increases the risk of human error and subjective interpretation. Moreover, inconsistencies in documentation, fragmented data across systems, and variability in clinical terminology pose significant barriers to efficient and standardized screening. These challenges contribute to delays in recruitment, potential misclassification of candidates, and reduced overall study efficiency. In this context, the advent of advanced artificial intelligence (AI) systems might offer promising solutions to improve trial screening workflows. Large language models offer a versatile, infrastructure-independent solution capable of interpreting unstructured clinical information. In recent years, various natural language processing (NLP) systems including International Business Machines (IBM) Watson Clinical Trial Matching, CogStack, and the Automated Clinical Trial Eligibility Screener (ACTES), have been evaluated for their suitability in clinical trial screening.^16^ While NLP-based systems have been shown to reduce manual workload and improve time efficiency, their utility remains limited by variable accuracy and a lack of robust validation in real-world settings.^16^ Chatbot generative pretrained transformer version 4.0 (ChatGPT-4.0), developed by OpenAI, represents a significant leap forward in NLP technologies.^17,18^ Built on a transformer-based architecture, this multimodal model is designed to process both image and text inputs, to generate human-like text and thus facilitate complex conversation dynamics.^17^ Research has demonstrated that ChatGPT-4.0 is not only capable of successfully completing medical exams but has also been extensively explored for its applicability in clinical practice.^18–23^

The aim of the present study is to evaluate the potential of AI, based on the example of ChatGPT-4.0, in identifying suitable study participants among patients with breast cancer, utilizing data from interdisciplinary tumor board presentations. Integration of remote technologies in the screening process could not only alleviate the burden on clinical sites and accelerate achieving recruitment goals, but may also improve access to clinical trials for patients.

## Methods

### Patient cohort

All patients with breast findings discussed in the interdisciplinary tumor board (ITB) of the Department of Gynecology and Obstetrics at the University Hospital Erlangen (Erlangen, Germany) in January 2024 were included in this study. Written informed consent was obtained from each patient prior to participation. The cohort contained patients with diverse characteristics regarding age, gender, comorbidities, and tumor-specific parameters. Benign findings were not excluded. A total of 124 suitable patients were identified.

### Pretraining of ChatGPT

**Figure 1.**
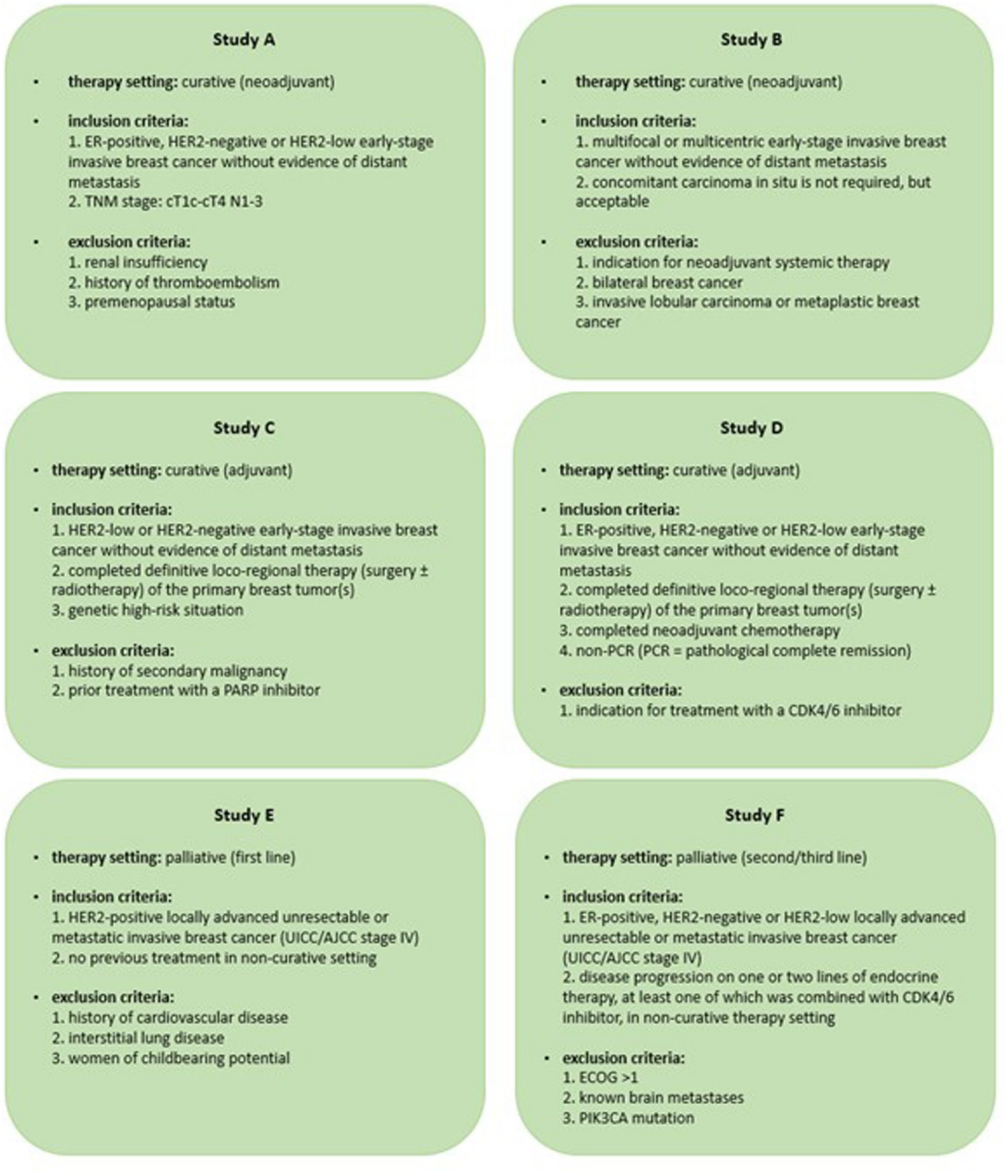
Fictitious study protocols A to -F.

ChatGPT-4.0 was trained on six fictitious study protocols for patients with breast cancer, mimicking real-world clinical trial scenarios (Figure 1). Two study protocols were developed by the research team for each treatment situation: neoadjuvant (studies A and B), adjuvant (studies C and D), and palliative (studies E and F). Each protocol included five eligibility criteria, closely modeled on the structure and rationale of contemporary breast oncology trials. In addition to tumor characteristics such as hormone receptor status, human epidermal growth factor receptor 2 (HER2) expression, molecular markers, and TNM classification, previous cancer therapies were also considered. Additionally, patient-related parameters such as ECOG (Eastern Co-operative Oncology Group) performance status, comorbidities, menopausal status, and genetic risk factors were integrated into the analysis. Although the dataset of the present analysis included patients with both malignant and benign breast findings, all fictitious study protocols were designed exclusively for malignant disease. Consequently, a key objective of the screening task was for both the AI and the control group to correctly exclude patients with benign diagnoses as ineligible.

For the purpose of pretraining, ChatGPT-4.0 was provided with information on the respective study setting as well as all inclusion and exclusion criteria.

### Methodology of data collection

Anonymized data from 124 tumor board registrations from January 2024 were submitted to the AI with the question of whether the eligibility criteria for the pretrained study protocols are met. For a patient to qualify for a study, all inclusion criteria had to be fulfilled in the absence of any exclusion criteria. The data was entered following a predefined standardized input protocol (see Table 1), derived from the tumor board registration format routinely applied at the Department of Gynecology and Obstetrics at University Hospital Erlangen. Patient-identifying information such as name, birth date, contact information, provenance, and attending physicians were explicitly excluded from the query. Additionally, to ensure anonymization, the patient's exact age was not specified. ChatGPT-4.0 was only informed whether the patient was above or below 55 years of age. Precise genetic findings were likewise omitted, with the only information provided being whether there was an elevated familial risk for malignancies. PIK3CA (phosphatidylinositol-4,5-bisphosphate 3-kinase catalytic subunit alpha) and ESR1 (estrogen receptor 1) mutations, if present, were also not disclosed in detail. Parallel to the data review by ChatGPT-4.0, a control group consisting of two board-certified gynecological oncologists and two assistant physicians with less than 1 year of professional experience assessed the patients’ eligibility for study participation. The physicians were presented with the identical standardized input as the AI, including both patient-specific information and the complete details of each study protocol.

**Table 1.** Standardized protocol for AI data submission.

age of the patient (<55 years/ ≥ 55 years)
ECOG status
Menopausal status
Underlying disease with histology, clinical stage, and localization of metastasis (if applicable)
Reason for current tumor board presentation
Tumor-related medical history in chronological order, including previous therapies
Staging examinations and tumor markers (if applicable)
Secondary diagnoses, allergies, and previous surgeries
Additional diagnostics: PIK3CA mutation status (mutation/ wild-type), PD-L1 status, ESR1 mutation status (mutation/ wild-type), genetic high-risk situation (yes/no)

ESR1: estrogen receptor 1; ECOG: Eastern Co-operative Oncology Group; PD-L1: Programmed death-ligand 1; PIK3CA: phosphatidylinositol-4,5-bisphosphate 3-kinase catalytic subunit alpha.

### Data review and statistical analysis

The performance of the AI and the medical professionals was evaluated using an expert-validated eligibility benchmark as a reference standard for comparison. The reference standard was established prior to analysis by two independent board-certified physicians through systematic application of the inclusion and exclusion criteria defined in the six study protocols to the study cohort. To ensure the accuracy of the reference standard, after comparing it with the responses from ChatGPT-4.0 and the control group, any deviation from the expert-validated eligibility benchmark was re-evaluated by the two independent physicians. Sensitivity and specificity each with a 95% confidence interval (CI) were calculated for the AI as well as for each member of the control group. The Clopper-Pearson method was used to calculate the CIs. Calculations were carried out using the R system for statistical computing (version 4.5.1; R Development Core Team, Vienna, Austria, 2025).

## Results

### Neoadjuvant study protocols (studies A and B)

**Figure 2.**
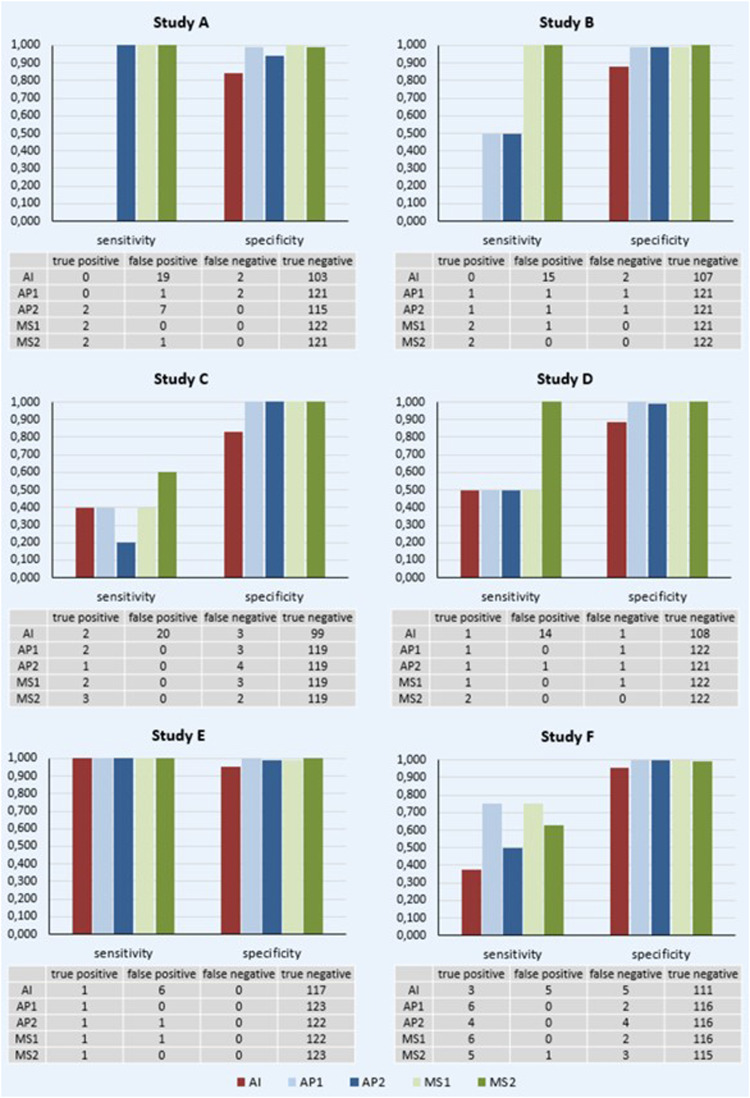
Performance of the artificial intelligence (AI), assistant physicians with less than 1 year of professional experience (AP) and medical specialists for gynecology and obstetrics (MS) with regard to patient identification for study protocols A to F.

According to the expert-validated eligibility benchmark, there were 2 out of 124 patients meeting the criteria for study A. ChatGPT-4.0 failed to identify any true positive cases and falsely marked 19 patients as eligible (sensitivity 0.0%, 95% CI: 0.0%–84.2%, specificity 84.4%, 95% CI: 76.8%–90.4%; Figure 2). Both medical specialists (MS1 and MS2) demonstrated better precision, with MS1 accurately assessing all 124 patients (2 true positives, 122 true negatives; sensitivity 100.0%, 95% CI: 15.8%–100.0%; specificity 100.0%, 95% CI: 97.0%–100.0%) and MS2 committing only a single error (2 true positives, 1 false positive, 121 true negatives; sensitivity 100.0%, 95% CI: 15.8%–100.0%; specificity 99.2%; 95% CI: 95.5%–100.0%; Figure 2). The evaluation of the assistant physicians’ (AP1 and AP2) performance revealed a higher frequency of incorrect decisions compared to the medical specialists (Figure 2). AP2 accurately identified both eligible patients (2 true positives; sensitivity 100.0%, 95% CI: 15.8%–100.0%) but misclassified seven others (7 false positives; specificity 94.3%, 95% CI: 88.5%–97.7%), while AP1 overlooked the two eligible patients (2 false negatives; sensitivity 0.0%, 95% CI: 0.0%–84.2%), albeit with a lower rate of false positives, recording only one instance (specificity 99.2%, 95% CI: 95.5%–100.0%).

For study B, there were also 2 out of 124 patients who met the inclusion and exclusion criteria. ChatGPT-4.0 again encountered difficulties, exhibiting a substantial number of false positives (n = 15; specificity 87.7%, 95% CI: 80.5%–93.0%) and failing to detect the two eligible study participants (sensitivity 0.0%, 95% CI: 0.0%–84.2%; Figure 2). AP1 and AP2 both accurately classified only one patient as suitable for study B (sensitivity 50.0%, 95% CI: 1.3%–98.7%), though recorded high specificity of 99.2% (95% CI: 95.5%–100.0%). MS1 and MS2 both exhibited superior sensitivity by accurately identifying two true positives (MS1 and MS2: sensitivity 100.0%, 95% CI: 15.8%–100.0%) and maintained high specificity with zero and one false positive, respectively (MS1: specificity 99.2%, 95% CI: 95.5%–100.0%; MS2: specificity 100.0%, 95% CI: 97.0%–100.0%; Figure 2).

### Adjuvant study protocols (studies C and D)

The analysis of studies C and D revealed significant deficiencies, particularly in sensitivity across almost all evaluators, including ChatGPT-4.0, assistant physicians, and medical specialists. In accordance with the expert-validated eligibility benchmark, among the 124 tumor board registrations, 5 participants were suitable for study C and 2 for study D. Only MS2 achieved sensitivity rates exceeding 50.0% for both study protocols (sensitivity of 60.0% (95% CI: 14.7%–94.7%) for study C and 100.0% for study D (95% CI: 15.8%–100.0%), Figure 2). The remaining physicians demonstrated notably poorer performance, with sensitivities ranging from 20.0% (95% CI: 0.5%–71.6%) to 40.0% (95% CI: 5.3%–85.3%) for study protocol C and an average sensitivity of 50.0% (95% CI: 1.3%–98.7%) for study protocol D (Figure 2). ChatGPT-4.0 achieved sensitivities of 40.0% (95% CI: 5.3%–85.3%, study C) and 50.0% (95% CI: 1.3%–98.7%, study D; Figure 2). Despite the substantial number of eligible patients being overlooked, the frequency of false positives was low across all assessments carried out by medical professionals, with only one false positive recorded by AP2 in study D, indicating high specificity (AP1, MS1, and MS2: specificity 100.0% for study C (95% CI: 96.9%–100.0%) and D (95% CI: 97.0%–100.0%); AP2: specificity 100.0% (95% CI: 96.9%–100.0%) for study C and 99.2% (95% CI: 95.5%–100.0%) for study D). In contrast, the AI system exhibited markedly poorer performance, with 20 and 14 false positives in studies C and D, respectively (specificity 83.2% (95% CI: 75.2%–89.4%) for study C and 88.5% (95% CI: 81.5%–93.6%) for study D; Figure 2).

### Study protocols for palliative setting/advanced stage (studies E and F)

For study E, the highest sensitivity and specificity values were recorded across all study protocols (Figure 2). All medical professionals and ChatGPT-4.0 successfully identified the patient eligible for study E (AI, AP1, AP2, MS1, and MS2: sensitivity 100.0%, 95% CI: 2.5%–100.0%). Despite a low frequency of false positives overall, a distinct disparity persisted between the physicians and the AI (Figure 2). ChatGPT-4.0 recurrently misclassified patients as eligible, resulting in six false positives (specificity 95.1%, 95% CI: 89.7%–98.2%). In contrast, only one AP and one MS each incorrectly identified a single patient as suitable (AP2 and MS1: specificity 99.2%, 95% CI: 95.6%–100.0%; AP1 and MS2: specificity 100.0%, 95% CI: 97.0%–100.0%). Screening for the study scenario F resulted in high specificity rates among all evaluators, with only five patients incorrectly defined as eligible by ChatGPT-4.0 and only one false positive decision in the group of medical professionals (AI: specificity 95.7%, 95% CI: 90.2%–98.6%; AP1, AP2, and MS1: specificity 100.0%, 95% CI: 96.9%–100.0%; MS2: specificity 99.1%, 95% CI: 95.3%–100.0%). Analyzing sensitivity, a different picture emerges. According to the expert-validated eligibility benchmark, eight patients were suitable for participation in study F. Both the AI and the medical professionals overlooked several candidates who fulfilled the eligibility criteria for the study (Figure 2). Sensitivity values varied widely, with the AI achieving only 37.5% sensitivity (95% CI: 8.5%–75.5%), while AP1 and MS1 each reached a sensitivity of 75.0% (95% CI: 34.9%–96.8%; Figure 2).

### Summary of results

Overall, among 124 tumor board registrations, 18 patients were suitable for 1 study and 1 patient qualified for 2 trials. Both the AI and the control group reliably identified individuals who were ineligible for study participation (high specificity). However, substantial disparities were evident in terms of sensitivity. In the context of the neoadjuvant breast cancer study protocols, ChatGPT-4.0 proved absolutely ineffective at screening for suitable patients. The medical professionals performed better on average, but partly showed very inhomogeneous results regarding sensitivity, with particularly poor values for study C. Interestingly, 18 out of 19 potential study participants among the 124 tumor board registrations were correctly identified by at least 1 physician.

## Discussion

The results of this study underscore the complexities and challenges inherent in integrating AI into clinical trial screening processes. Despite advancements in machine learning and AI, these technologies often do not match the diagnostic accuracy of experienced medical professionals. In the present analysis, ChatGPT-4.0 consistently demonstrated high specificity with values ranging from 83.2% to 95.7%, identifying patients unsuitable for study participation in numerous cases. However, the AI displayed marked variability in sensitivity depending on the study protocol, with sensitivity scores spanning from 0.0% to 100.0% across different studies. This volatility indicates that ChatGPT-4.0 cannot be reliably used to screen patients who fulfill the eligibility criteria for breast cancer clinical trials. Interestingly, the present analysis revealed deficiencies not only in the performance of ChatGPT-4.0 but also among medical professionals. Regarding specificity, all physicians showed high reliability, with specificity levels ranging from 94.3% to 100% across all studies, consistently exceeding those observed in the AI. This suggests that medical professionals were generally adept at identifying individuals who were not appropriate candidates for study participation. Conversely, considerable variability was evident both between and within groups of medical specialists and attending physicians, with respect to sensitivity. For example, in study A, sensitivity varied dramatically, with AP1 recording 0.0% sensitivity, while AP2 achieved 100.0% sensitivity. The variance in performance among assistant physicians may reflect differences in experience or familiarity with specific eligibility criteria, highlighting the importance of continuous training and education in clinical research methodologies. The on average superior performance of medical specialists emphasizes the value of expertise and specialized training for achieving high diagnostic accuracy, which is crucial in the context of clinical trials, where efficient screening processes enhance cost efficiency, save time for healthcare providers, expedite therapy initiation, may reduce psychological stress for patients, and improve their access to study treatment. Despite the study-dependent low sensitivity scores of individual physicians, the medical professionals exhibited good overall performance. 18 out of 19 potential study participants among the 124 tumor board registrations were correctly identified by at least 1 physician, highlighting the superiority of team-based compared to individual decision making.

A limitation of the present analysis is the small sample size of only 124 tumor board registrations, where an incorrect assessment of even a single patient significantly impacts the calculation of specificity and sensitivity. Especially the small number of eligible patients is reflected in the wide confidence intervals for the sensitivities. While the current findings provide valuable insights, further studies should aim to include larger, multicenter cohorts to strengthen the external validity and confirm the reliability of the results beyond the constraints of a single-center sample. The control group in this study comprised two board-certified gynecologic oncologists and two assistant physicians with less than 1 year of experience, reflecting a realistic range of clinical expertise. The limited number of reviewers and variation in clinical experience are acknowledged limitations of the present analysis. However, the inclusion of both experienced specialists and early-career physicians offers a balanced perspective and reflects the diversity of clinical decision making found in everyday practice. Importantly, the superior performance of the human control group compared to the AI model reinforces the validity of their assessments despite these structural limitations.

The use of AI for patient identification also raises regulatory and ethical considerations. Compliance with the General Data Protection Regulation (GDPR) in the European Union is essential when processing sensitive health data. Ensuring patient confidentiality, data integrity and rigorous data governance is indispensable to prevent re-identification risks when implementing AI-assisted trial screening. To ensure data protection, patient information was provided to both ChatGPT-4.0 and the control group of medical professionals using standardized input protocols derived from the tumor board registration format routinely applied at the Department of Gynecology and Obstetrics at University Hospital Erlangen. The AI and the medical professionals received consistent clinical data, including ECOG performance status, menopausal status, diagnosis with histological subtype, TNM classification, tumor-related medical history, relevant comorbidities, and documented allergies. In addition, the presence of hereditary cancer syndromes, PIK3CA or ESR1 mutations, and PD-L1 expression status were indicated. However, specific nomenclature of molecular variants was intentionally omitted to minimize the risk of patient re-identification. Likewise, the exact patient age was not disclosed; instead, a categorical variable indicating whether the patient was under 55 or 55 years and older was provided. Accordingly, the design of the fictitious study protocols was limited to five eligibility criteria per protocol in order to ensure compatibility with the level of detail provided by the de-identified and simplified patient data. It is acknowledged that this data simplification constitutes a key limitation of the study. Nevertheless, this approach was deliberately chosen to uphold the highest standards of data privacy and ethical responsibility in the handling of sensitive patient information. In real-life clinical settings, patient data that have not been preprocessed need to be analyzed during study screenings, potentially containing more irrelevant information, and sometimes requiring further interpretation. Continuing with the example of genetic predisposition, if a germline variant is present, it is necessary to determine whether there is a familial high-risk situation. Given the standardized and - for data security purposes - generalized patient information for data entry and the limited number of eligibility criteria used in this study, it remains uncertain whether ChatGPT-4.0 could effectively review complex and comprehensive patient records, accurately interpret the raw data and systematically evaluate a broad set of intricate eligibility requirements. Future research should investigate the potential of ChatGPT-4.0 for practical use in real-world clinical trial screening processes.

A fundamental challenge in employing ChatGPT-4.0 is the reliance of AI responses on quality of provided data. It has also been demonstrated that ChatGPT frequently fails to give any or adequate source attribution for its output information.^24^ In the medical environment in particular, however, the data credibility on which treatment decisions are based is indispensable. Furthermore, incorporating ChatGPT into healthcare facilities raises ethical concerns regarding patient privacy. It is imperative to carefully check all information supplied to the AI in advance to prevent data security violations. Responsible management of medical data and accurate interpretation of AI-provided information are critical requirements for deploying AI solutions in clinical environments.

The present study has demonstrated that team performance exceeds individual performance in breast cancer clinical trial screening. Given that ChatGPT-4.0 did not meet the standards of medical professionals, the focus shifts from replacing physicians to effectively integrating AI into teams for cost and time efficient data processing. Our study showed high levels of specificity for ChatGPT-4.0, though it fell short of physician expertise. While general-purpose language models like ChatGPT-4.0 are versatile and easy to use, they are not specifically designed for clinical trial recruitment. Unlike dedicated systems, ChatGPT-4.0 lacks built-in knowledge of clinical classifications, structured eligibility rules, or validated trial criteria. Continued model refinement through domain-specific training, access to larger and more heterogeneous datasets, and the integration of multimodal inputs, such as imaging, laboratory values, and clinical narratives, may allow AI systems to process complex patient profiles more effectively and holds considerable potential to overcome current limitations. It is conceivable that advanced architectures of large language models such as generative pretrained transformer version 5.0 (GPT-5) by OpenAI or domain-specific multimodal models like Med-Gemini by Google could perform better and thus be suitably applied for preselection.^25^ A potential integration pathway involves the deployment of AI-based prescreening modules within electronic health record systems. These tools could systematically extract and evaluate structured and unstructured patient data against predefined eligibility criteria, generating a shortlist of candidates accompanied by transparent inclusion and exclusion summaries for clinical review. Targeted rule-based submodules - for example, automated exclusion based on contraindications, missing diagnostics, or comorbidities - may further reduce manual screening demands. Embedding such AI components as supportive layers under human supervision could streamline the screening process while maintaining clinical oversight and ensuring regulatory compliance.

## Conclusion

The study underscores that although ChatGPT-4.0 can rapidly handle extensive datasets, it still falls short of the detailed judgment that physicians exercise when evaluating patients for clinical trials. In its current form, ChatGPT-4.0 cannot be recommended for patient identification for breast cancer clinical trials. However, continued refinement of AI models may enhance their capacity to interpret complex patient data and offers promising potential to mitigate existing limitations. Moreover, to optimize participant recruitment, the deployment of a multiprofessional team is essential.

## Supplemental Material

sj-docx-1-dhj-10.1177_20552076251389325 - Supplemental material for Evaluating the potential of ChatGPT for patient identification in clinical breast cancer trials Supplemental material, sj-docx-1-dhj-10.1177_20552076251389325 for Evaluating the potential of ChatGPT for patient identification in clinical breast cancer trials by Annika Krückel, Peter A Fasching, Oliver Schleicher, Julia Gocke, Lena Brückner, Katharina Seitz, Lothar Häberle, Felix Heindl, Carolin C Hack, Matthias W Beckmann and Julius Emons in DIGITAL HEALTH

sj-doc-2-dhj-10.1177_20552076251389325 - Supplemental material for Evaluating the potential of ChatGPT for patient identification in clinical breast cancer trials Supplemental material, sj-doc-2-dhj-10.1177_20552076251389325 for Evaluating the potential of ChatGPT for patient identification in clinical breast cancer trials by Annika Krückel, Peter A Fasching, Oliver Schleicher, Julia Gocke, Lena Brückner, Katharina Seitz, Lothar Häberle, Felix Heindl, Carolin C Hack, Matthias W Beckmann and Julius Emons in DIGITAL HEALTH

## Abbreviations

ACTES: Automated Clinical Trial Eligibility Screener
AI: Artificial intelligence
AJCC: American Joint Committee on Cancer
Akt: protein kinase B
AP: Assistant physician
CDK4/6: Cyclin-dependent kinases 4 and 6
ChatGPT-4.0: Chatbot generative pretrained transformer version 4.0
ECOG: Eastern Co-operative Oncology Group
ESR1: Estrogen receptor 1
GDPR: General Data Protection Regulation
HER2: Human epidermal growth factor receptor 2
IBM: International Business Machines
ITB: Interdisciplinary tumor board
MS: Medical specialists for gynecology and obstetrics
PARP: poly(adenosine diphosphate [ADP]-ribose) polymerase
PCR: Pathological complete remission
PD-L1: Programmed death-ligand 1
PI3K: Phosphatidylinositol 3-kinase
PIK3CA: Phosphatidylinositol-4,5-bisphosphate 3-kinase catalytic subunit alpha
SERD: Selective estrogen receptor degrader
TNM: Tumor node metastasis
UICC: Union for International Cancer Control
