# Supplementary material for: Evaluating the potential of ChatGPT for patient identification in clinical breast cancer trials
Source: Digit Health. 2025 Nov 20;11:20552076251389325. doi: 10.1177/20552076251389325 (PMC12639235; doi:10.1177/20552076251389325)
Supplement: sj-docx-1-dhj-10.1177_20552076251389325 - Supplemental material for Evaluating the potential of ChatGPT for patient identification in clinical breast cancer trials [file sj-docx-1-dhj-10.1177_20552076251389325.docx]

**Study protocols**

**Study A**

- **therapy setting:** curative (neoadjuvant)
- **inclusion criteria:**

1. ER-positive, HER2-negative or HER2-low early-stage invasive breast cancer without evidence of distant metastasis
2. TNM stage: cT1c-cT4 N1-3

- **exclusion criteria:**

1. renal insufficiency
2. history of thromboembolism
3. premenopausal status

**Study B**

- **therapy setting:** curative (neoadjuvant)
- **inclusion criteria:**

1. multifocal or multicentric early-stage invasive breast cancer without evidence of distant metastasis
2. concomitant carcinoma in situ is not required, but acceptable

- **exclusion criteria:**

1. indication for neoadjuvant systemic therapy
2. bilateral breast cancer
3. invasive lobular carcinoma or metaplastic breast cancer

**Study C**

- **therapy setting:** curative (adjuvant)
- **inclusion criteria:**

1. HER2-low or HER2-negative early-stage invasive breast cancer without evidence of distant metastasis
2. completed definitive loco-regional therapy (surgery ± radiotherapy) of the primary breast tumor(s)
3. genetic high-risk situation

- **exclusion criteria:**

1. history of secondary malignancy
2. prior treatment with a PARP inhibitor

**Study D**

- **therapy setting:** curative (adjuvant)
- **inclusion criteria:**

1. ER-positive, HER2-negative or HER2-low early-stage invasive breast cancer without evidence of distant metastasis
2. completed definitive loco-regional therapy (surgery ± radiotherapy) of the primary breast tumor(s)
3. completed neoadjuvant chemotherapy
4. non-PCR (PCR = pathological complete remission)

- **exclusion criteria:**

1. indication for treatment with a CDK4/6 inhibitor

**Study E**

- **therapy setting:** palliative (first line)
- **inclusion criteria:**

1. HER2-positive locally advanced unresectable or metastatic invasive breast cancer (UICC/AJCC stage IV)
2. no previous treatment in non-curative setting

- **exclusion criteria:**

1. history of cardiovascular disease
2. interstitial lung disease
3. women of childbearing potential

**Study F**

- **therapy setting:** palliative (second/third line)
- **inclusion criteria:**

1. ER-positive, HER2-negative or HER2-low locally advanced unresectable or metastatic invasive breast cancer (UICC/AJCC stage IV)
2. disease progression on one or two lines of endocrine therapy, at least one of which was combined with CDK4/6 inhibitor, in non-curative therapy setting

- **exclusion criteria:**

1. ECOG >1
2. known brain metastases
3. PIK3CA mutation
