# Supplementary material for: Evaluating the potential of ChatGPT for patient identification in clinical breast cancer trials
Source: Digit Health. 2025 Nov 20;11:20552076251389325. doi: 10.1177/20552076251389325 (PMC12639235; doi:10.1177/20552076251389325)
Supplement: sj-doc-2-dhj-10.1177_20552076251389325 - Supplemental material for Evaluating the potential of ChatGPT for patient identification in clinical breast cancer trials [file sj-doc-2-dhj-10.1177_20552076251389325.doc]

STROBE Statement

|  | Item No | Recommendation | Response |
| --- | --- | --- | --- |
| **Title and abstract** | 1 | (*a*) Indicate the study’s design with a commonly used term in the title or the abstract | The abstract explicitly states that this is a cross-sectional study. “This cross-sectional study evaluates the potential of artificial intelligence (AI), based on the example of ChatGPT-4.0, in identifying suitable study participants among breast cancer patients, utilizing real-world tumor board data.” |
|  |  | (*b*) Provide in the abstract an informative and balanced summary of what was done and what was found | The abstract summarizes objective (evaluating the potential of ChatGPT-4.0 for patient identification in breast cancer clinical trials), methods (training of ChatGPT-4.0 on fictious study protocols; study screening by AI and clinician control group; benchmarking of screening results against an expert-validated reference standard; calculation of sensitivity and specificity), results (performance of ChatGPT-4.0 versus medical professionals, impact of team-based decision-making), and conclusions on AI utility. |
| Introduction | | |  |
| Background/rationale | 2 | Explain the scientific background and rationale for the investigation being reported | The introduction describes challenges in screening patients for clinical breast cancer trials, and the potential role of ChatGPT-4.0 in reducing workload and errors. AI systems other than ChatGPT-4.0, including IBM Watson Clinical Trial Matching, CogStack, and ACTES, are also discussed to contextualize the study withing existing AI approaches for trial recruitment. |
| Objectives | 3 | State specific objectives, including any prespecified hypotheses | Objective: to evaluate the potential of ChatGPT-4.0 to identify eligible patients for clinical breast cancer trials using real-world tumor board data |
| Methods | | |  |
| Study design | 4 | Present key elements of study design early in the paper | The cross-sectional design is described at the beginning of the Methods section; presented key elements include training of ChatGPT-4.0 on fictious study protocols; presentation of the data to ChatGPT-4.0 and the control group of medical professionals; study screening by AI and clinician control group; benchmarking of screening results against an expert-validated reference standard; calculation of sensitivity and specificity |
| Setting | 5 | Describe the setting, locations, and relevant dates, including periods of recruitment, exposure, follow-up, and data collection | Setting: Department of Gynecology and Obstetrics, University Hospital Erlangen, Germany; data collected from January 2024 tumor board registrations. |
| Participants | 6 | (*a*) Give the eligibility criteria, and the sources and methods of selection of participants | 124 tumor board registrations in the Department of Gynecology and Obstetrics, University Hospital Erlangen, Germany, from January 2024 included; all patients who provided written informed consent; both malignant and benign breast findings included; all data anonymized |
| Variables | 7 | Clearly define all outcomes, exposures, predictors, potential confounders, and effect modifiers. Give diagnostic criteria, if applicable | Outcome: eligibility for clinical trial participation; exposures: assessment by ChatGPT-4.0 or by clinicians |
| Data sources/ measurement | 8* | For each variable of interest, give sources of data and details of methods of assessment (measurement). Describe comparability of assessment methods if there is more than one group | Patient data extracted from tumor board registrations; standardized input used for both AI and clinicians; expert-validated benchmark used for comparison |
| Bias | 9 | Describe any efforts to address potential sources of bias | Standardized data input, inclusion of clinicians with different levels of experience, anonymized patient information, and use of expert benchmark reduce bias |
| Study size | 10 | Explain how the study size was arrived at | All 124 tumor board registrations from the specified month included who provided written informed consent; no formal sample size calculation performed |
| Quantitative variables | 11 | Explain how quantitative variables were handled in the analyses. If applicable, describe which groupings were chosen and why | Age categorized as <55 or ≥55; other variables reported as categorical or binary; simplification noted as limitation |
| Statistical methods | 12 | (*a*) Describe all statistical methods, including those used to control for confounding | Sensitivity and specificity each with a 95% confidence interval (CI) were calculated for the AI as well as for each member of the control group; Clopper-Pearson method was used to calculate the Cis; calculations were carried out using the R system for statistical computing (version 4.5.1; R Development Core Team, Vienna, Austria, 2025); subgroup analyses by study type (neoadjuvant, adjuvant, palliative); no missing data |
|  |  | (*b*) Describe any methods used to examine subgroups and interactions |  |
|  |  | (*c*) Explain how missing data were addressed |  |
|  |  | (*d*) If applicable, describe analytical methods taking account of sampling strategy |  |
|  |  | (*e*) Describe any sensitivity analyses |  |
| Results | | |  |
| Participants | 13* | (a) Report numbers of individuals at each stage of study—eg numbers potentially eligible, examined for eligibility, confirmed eligible, included in the study, completing follow-up, and analysed | 124 registrations assessed; no cases excluded from analysis |
|  |  | (b) Give reasons for non-participation at each stage |  |
|  |  | (c) Consider use of a flow diagram |  |
| Descriptive data | 14* | (a) Give characteristics of study participants (eg demographic, clinical, social) and information on exposures and potential confounders | Detailed patient characteristics (e.g., demographics, clinical data) are not presented in the manuscript because they are not essential for the study’s main objective, which is to evaluate the performance of ChatGPT-4.0 versus clinical experts. To protect patient privacy, only aggregated and anonymized data were used. Detailed patient-level data can be made available upon justified request. |
|  |  | (b) Indicate number of participants with missing data for each variable of interest | Not applicable; all data used for AI and clinician assessments were complete, and no missing values affected the study analysis. |
| Outcome data | 15* | Report numbers of outcome events or summary measures | Number of eligible patients (19 of 124) reported; AI and clinician performance summarized |
| Main results | 16 | (*a*) Give unadjusted estimates and, if applicable, confounder-adjusted estimates and their precision (e.g., 95% confidence interval). Make clear which confounders were adjusted for and why they were included | Sensitivity and specificity were calculated for ChatGPT-4.0 and each member of the clinician control group, using an expert-validated reference standard. 95% confidence intervals were reported for all estimates (Clopper-Pearson method). No adjustments for confounders were performed, as the study design did not include exposure-outcome comparisons. |
|  |  | (*b*) Report category boundaries when continuous variables were categorized | Continuous variables relevant for eligibility, such as age, were categorized (<55 years or ≥55 years). Other patient characteristics were provided in categorical or binary form for AI and clinician assessment. Data simplification was noted as limitation. |
|  |  | (*c*) If relevant, consider translating estimates of relative risk into absolute risk for a meaningful time period | Not applicable, as the study does not report time-to-event outcomes or relative risks; the analysis focuses on performance measures (sensitivity and specificity) for patient identification at a single time point. |
| Other analyses | 17 | Report other analyses done - e.g. analyses of subgroups and interactions, and sensitivity analyses | Subgroup analyses were performed for each type of study protocol (neoadjuvant, adjuvant, palliative) to assess performance differences of AI and clinicians. Team-based performance was evaluated by determining whether at least one physician correctly identified eligible patients. No additional sensitivity analyses were required, as all data were complete and standardized. |
| Discussion | | |  |
| Key results | 18 | Summarise key results with reference to study objectives | AI showed high specificity but variable sensitivity; clinicians performed better on average; team-based approach most effective |
| Limitations | 19 | Discuss limitations of the study, taking into account sources of potential bias or imprecision. Discuss both direction and magnitude of any potential bias | Small sample size, simplified patient data, single-center study, AI limitations, heterogeneous clinician experience |
| Interpretation | 20 | Give a cautious overall interpretation of results considering objectives, limitations, multiplicity of analyses, results from similar studies, and other relevant evidence | The results of this study are interpreted with caution in light of the study objectives and limitations. ChatGPT-4.0 demonstrated high specificity but variable and often low sensitivity, particularly in neoadjuvant and adjuvant study scenarios. Clinicians generally outperformed the AI, although performance varied across individual evaluators, highlighting the importance of expertise and team-based decision-making. The study acknowledges limitations such as a small, single-center cohort, simplified patient data, and the lack of longitudinal follow-up. Comparisons with other AI systems, including IBM Watson Clinical Trial Matching, CogStack, and ACTES, contextualize these findings within the broader field of AI-assisted clinical trial recruitment, showing that general-purpose AI tools have potential but currently cannot replace expert human judgment. Overall, the study suggests that AI may serve as a supportive tool within a clinical team, but careful oversight and further model refinement are necessary before independent deployment in patient selection. |
| Generalisability | 21 | Discuss the generalisability (external validity) of the study results | It was discussed that findings may not generalize beyond single-center or simplified dataset; multicenter studies recommended. |
| Other information | | |  |
| Funding | 22 | Give the source of funding and the role of the funders for the present study and, if applicable, for the original study on which the present article is based | No funding; statement included in manuscript. |

*Give information separately for exposed and unexposed groups.

**Note:** An Explanation and Elaboration article discusses each checklist item and gives methodological background and published examples of transparent reporting. The STROBE checklist is best used in conjunction with this article (freely available on the Web sites of PLoS Medicine at http://www.plosmedicine.org/, Annals of Internal Medicine at http://www.annals.org/, and Epidemiology at http://www.epidem.com/). Information on the STROBE Initiative is available at www.strobe-statement.org.
